# Magnified Examination of Small Colorectal Polyps Using a Prototype Electronic Endoscope

**DOI:** 10.1155/DTE.6.77

**Published:** 2000

**Authors:** O. Katayama, K. Namiki, K. Iwakoshi, H. Fujita, H. Yasuhara, I. Ohi, S. Tomatsu

**Affiliations:** 1 Clinical Laboratory Saitamaken-Saiseikai Kurihashi Hospital Kouemon 714-6 Kurihashi-cho, Kitakatsusika-gun Saitama 349-1105 Japan; 2 Nasu Factory Toshiba Corporation Shimoishigami 1385 Ohtawara-shi Tochigi 324-0036 Japan; 3 Laboratory Tokyo Women's Medical University Daini Hospital Tokyo 116-0011 Japan

## Abstract

Magnifying electronic endoscopes are frequently used to evaluate the pit patterns of the colorectal mucosa, but such endoscopes suffer from a number of problems. For example, they tend to have long, hard tips and heavy controller sections. In addition, the magnified endoscopic images obtained are often quite coarse due to the small number of pixels in the charge-coupled device (CCD). As a result, at higher magnification ratios, the orientation of the field of view is easily lost. A newly developed prototype colorectal electronic endoscope (Toshiba Corporation, Tokyo) overcomes these problems. The length of the hard tip of the scope and the weight of the controller section are comparable to those of the TCE-3680MH (Toshiba Corporation). High-resolution magnified images can be obtained, because a 410,000-pixel CCD is employed. Two magnification methods are available, optical magnification and electronic zooming, permitting images to be magnified by a factor of up to 90–120 without losing the orientation of the field of view. This newly developed magnifying electronic endoscope was found to be very useful, allowing us to observe the pit patterns of the colorectal mucosa in 82 small colorectal polyps measuring 7 mm or less in diameter.


*Diagnostic and Therapeutic Endoscopy*, Vol. 6, pp. 77–82
Reprints available directly from the publisher
Photocopying permitted by license only

© 2000 OPA (Overseas Publishers Association) N.V.
Published by license under
the Harwood Academic Publishers imprint,
part of The Gordon and Breach Publishing Group.
Printed in Malaysia.

### Magnified Examination of Small Colorectal Polyps Using a Prototype Electronic Endoscope: Preliminary Experience

O. KATAYAMA[a,*], K. NAMIKI[a], K. IWAKOSHI[b], H. FUJITA[b], H. YASUHARA[b], I. OHI[c] and S. TOMATSU[c]

[a]*Clinical Laboratory, Saitamaken-Saiseikai Kurihashi Hospital, Kouemon 714-6, Kurihashi-cho, Kitakatsusika-gun, Saitama 349-1105, Japan;* [b]*Nasu Factory, Toshiba Corporation, Shimoishigami 1385, Ohtawara-shi, Tochigi 324-0036, Japan;* [c]*Laboratory Tokyo Women's Medical University Daini Hospital, Tokyo 116-0011, Japan*

*(Received 11 June 1999; Revised 29 July 1999; In final form 20 October 1999)*

**Magnifying electronic endoscopes are frequently used to evaluate the pit patterns of the colorectal mucosa, but such endoscopes suffer from a number of problems. For example, they tend to have long, hard tips and heavy controller sections. In addition, the magnified endoscopic images obtained are often quite coarse due to the small number of pixels in the charge-coupled device (CCD). As a result, at higher magnification ratios, the orientation of the field of view is easily lost. A newly developed prototype colorectal electronic endoscope (Toshiba Corporation, Tokyo) overcomes these problems. The length of the hard tip of the scope and the weight of the controller section are comparable to those of the TCE-3680MH (Toshiba Corporation). High-resolution magnified images can be obtained, because a 410,000-pixel CCD is employed. Two magnification methods are available, optical magnification and electronic zooming, permitting images to be magnified by a factor of up to 90–120 without losing the orientation of the field of view. This newly developed magnifying electronic endoscope was found to be very useful, allowing us to observe the pit patterns of the colorectal mucosa in 82 small colorectal polyps measuring 7 mm or less in diameter.**

*Keywords:* CCD, Colorectal polyp, Electronic endoscope, Magnified examination

### INTRODUCTION

Endoscopic therapy should always be performed based on the findings of high-quality endoscopic examination. Recently, it has become commonplace to employ magnified endoscopy to assess the need for endoscopic therapy and to select the appropriate therapeutic method. For example, the relationship between colorectal tumors and mucosal pit patterns has been reported in the literature [1]. Magnifying electronic endoscopes (zooming electronic endoscopes) are frequently

* Corresponding author. Tel.: +81-480-52-3611. Fax: +81-480-52-1348. E-mail: saisei@mx7.mesh.ne.jp.

employed to evaluate the pit patterns of the colorectal mucosa.

For the purpose of high-quality endoscopy, we have developed a prototype colorectal electronic endoscope with improved operability. This new scope provides bright, high-resolution images in conventional examinations and also permits the pit patterns of the colorectal mucosa to be examined with ease. In the present study, we evaluated the clinical usefulness of this scope, focusing on the examination of small colorectal polyps measuring 10 mm or less in diameter.

### MATERIALS AND METHODS

A prototype colorectal electronic endoscope (Toshiba Corporation, Tokyo) was used in this study. The external diameter of the tip of this scope is 13.2 mm, and the external diameter of the flexible section is 13.5 mm. The length of the hard tip and the weight of the controller section are comparable to those of a conventional electronic endoscope (TCE-3680MH, Toshiba Corporation, Tokyo) [2]. Since a 410,000-pixel CCD is employed, high-resolution magnified images can be obtained. The scope has a 140° angle of view in conventional examinations and a 90° angle of view in magnified examinations. The magnification ratio in the area of maximum resolution is 25 times (viewing distance: 3 mm) in conventional examinations and 60 times (viewing distance: 2 mm) in magnified examinations when images are displayed on a 14-inch TV monitor. In addition, images can be further magnified by a factor of 1.5–2.0 by using the electronic zooming function in combination [3], resulting in a maximum magnification ratio of 90 ($60 \times 1.5$) to 120 ($60 \times 2$) with a 14-inch TV monitor, without loss of orientation of the field of view. In order to avoid missing small lesions, we used the endoscope equipped with an oblique transparent hood (Obli-Clear, Top Corporation, Tokyo).

Lesions were washed with water via a dedicated water channel sprayed with 0.02% crystal violet [4], and then washed with water again. In order to ensure sufficient dyeing, lesions were sprayed with 0.02% crystal violet a second time and washed to remove excessive dye and mucus. Using this method, satisfactory dyeing of the mucosal pits can be achieved in most cases. If satisfactory dyeing is not obtained, the above procedures are repeated.

After the dyed lesion has been carefully examined at standard magnification, optical magnification is selected by sliding a lever provided at the bottom of the controller section of the scope. Then, using either a button on the controller section or a foot switch connected to the main body of endoscope, the electronic zooming function is actuated to zoom in by a factor of 1.5–2.0 to perform examination at higher magnification. Two basic methods are used for magnified examination using this scope: (1) standard examination → optical magnification → electronic zooming, and (2) standard examination → electronic zooming → optical magnification. The appropriate examination method is selected depending on the location of the lesion and intestinal movements.

We performed magnified examination for 82 lesions in 19 patients with small colorectal polyps measuring 7 mm or less in diameter between October 26 and 28, 1998 and between February 22 and March 3, 1999. Dyeing characteristics and pit patterns were compared against histopathological findings in resected specimens.

### RESULTS

Satisfactory dyeing was achieved for the evaluation of the pit patterns of the colorectal mucosa in all 82 lesions. These results indicate that magnified endoscopic examination is useful for predicting the histopathological characteristics of lesions.

### 1. Non-Tumorous Lesions

Forty-four of the 82 lesions were histopathologically identified as hyperplastic nodules or metaplastic polyps measuring 0.3–6.0 mm in diameter (mean diameter: 2.5 mm). In most lesions, regular stellate,

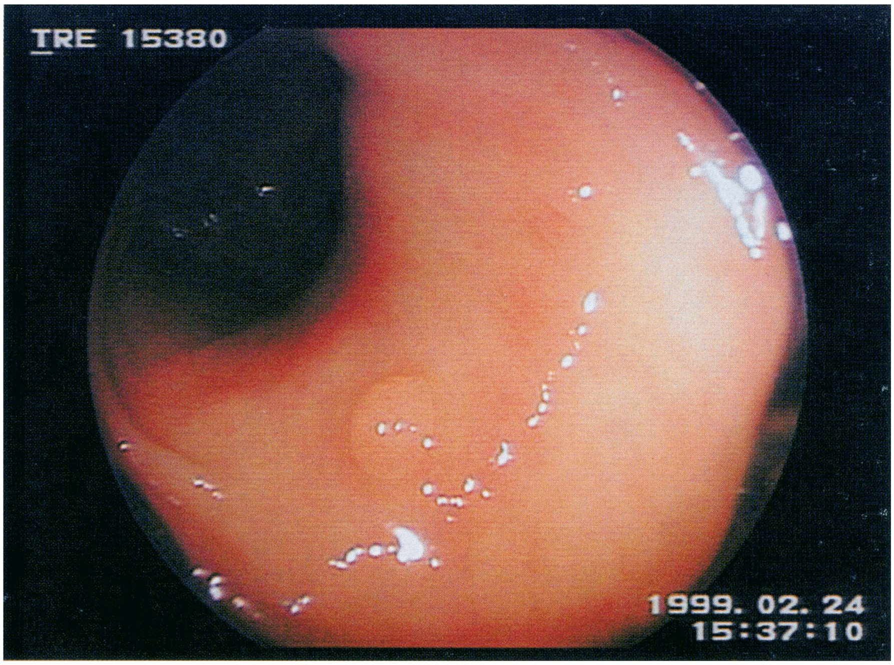

FIGURE 1 An endoscopic image of a small, 2.5 mm polyp in the transverse colon examined at normal magnification.

circular, or oval pit patterns were observed. In a few lesions, some areas showed a tubular pattern.

Figure 1 shows an endoscopic image of a small polyp measuring 2.5 mm in diameter in the transverse colon that was examined at normal magnification. After the lesion was dyed twice using crystal violet, it was carefully examined at normal magnification. Then, an optically magnified image was obtained by sliding the lever at the bottom of the controller section, and the image was further electronically zoomed (×2.0) using the foot switch connected to the endoscope. Regular oval pit patterns were observed (Fig. 2). Such lesions were histopathologically identified as non-tumorous hyperplastic nodules.

### 2. Adenomas

Of the 82 lesions evaluated, 38 were histopathologically identified as adenomas with mild to moderate atypia. No highly atypical adenomas, carcinomas in adenoma, or carcinomas were detected. All of these lesions measured between 1.0 and 7.0 mm in diameter (average diameter: 3.2 mm). The pit patterns of these lesions were tubular or partly oval, showing a regular arrangement.

Figure 3 shows an endoscopic image of a small, 1.5 mm polyp in the transverse colon examined at normal magnification. This lesion was dyed twice using crystal violet. An optically magnified image was obtained by sliding the lever at the bottom of the controller section of the scope, and an electronically zoomed image (×2.0) was then obtained by pressing the button on the controller section. Endoscopic examination showed a tubular and oval pit pattern with a regular arrangement (Fig. 4). The lesion was histopathologically identified as a tubular adenoma showing mild atypia.

### DISCUSSION

The history of colorectal endoscopy can be traced back to 1963, when Turell designed the first fiberscope for the examination of the colon [5]. Since that time, fiberscopes have evolved into

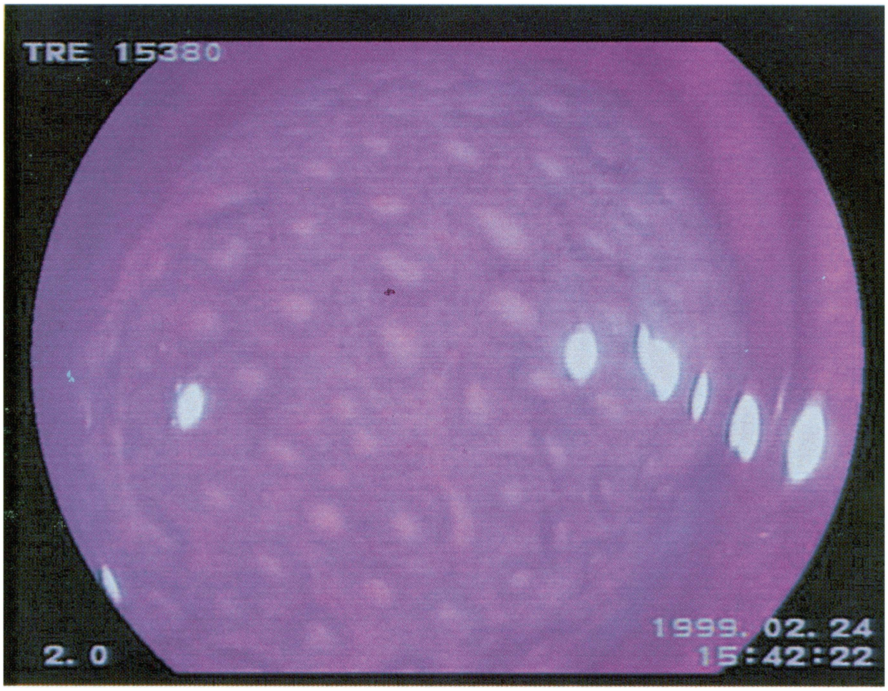

FIGURE 2 After the lesion was dyed twice using crystal violet, regular oval pit patterns were observed by optical magnification and electronic zooming (×2.0).

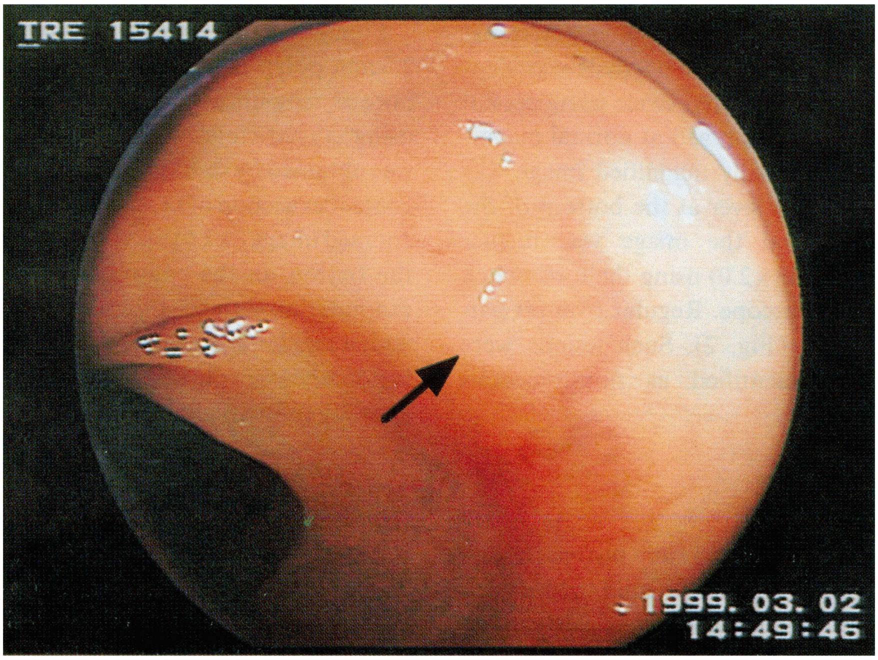

FIGURE 3 An endoscopic image of a small, 1.5 mm polyp in the transverse colon examined at normal magnification.

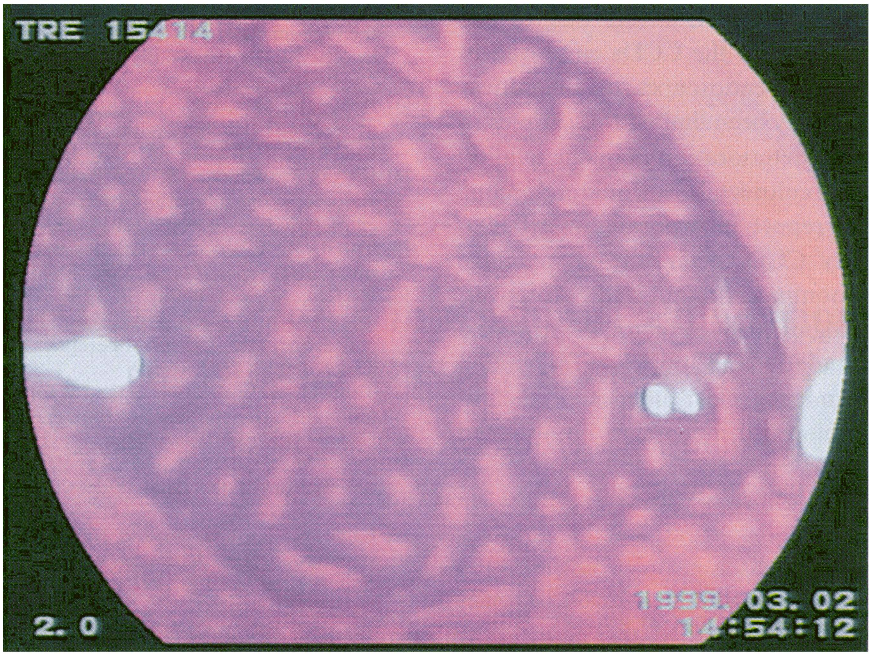

FIGURE 4 After the lesion was dyed twice using crystal violet, a tubular and oval pit pattern with a regular arrangement was observed by optical magnification and electronic zooming (×2.0).

electronic endoscopes [6]. This evolution in endoscope design has brought about major improvements in image quality. Factors that influence overall image quality include the objective lens, the charge-coupled device (CCD), the scope's operability and electronic processing capabilities, the operator's skill, the maintenance and operating condition of the device, and so on. In particular, the CCD is the most important factor determining the image quality of electronic endoscopes. In short, the larger the number of pixels in the CCD, the higher the endoscope's resolution. Since the prototype colorectal electronic endoscope used in the present study incorporates a 410,000-pixel, high-density CCD, which is limited only by the 525 scanning lines of a conventional TV monitor, high-resolution endoscopic images can be obtained, as shown by the representative images presented in this report.

Contact observation using a standard endoscope has also been used for the magnified endoscopic examination of the digestive tract. However, the magnification ratio that can be achieved using this method is too low to permit satisfactory observation of the pit patterns of the colorectal mucosa [7]. In addition, magnifying endoscopes with a narrow field of view are now available for such examinations. With regard to such endoscopes, one type has a fixed-focus lens, as represented by the magnifying fiberscope [8]. This type is seldom used in routine clinical practice because the target region is easily lost and operation tends to be difficult due to loss of perspective in the field of view. On the other hand, magnifying endoscopes with variable-focus lenses are now employed in routine clinical practice. Such scopes have been made possible, thanks to the development of magnifying electronic endoscopes, and have been reported to be particularly useful for the examination of the pit patterns of the colorectal mucosa [1,9].

The advantage of the electronic zooming function, which permits images to be zoomed on the TV display screen [3], is that there is less risk of losing the target region, since magnified images can be observed instantaneously. However, due to the small number of pixels in the CCDs employed in conventional electronic endoscopes, the magnification ratio has generally been limited to 1.5 times to avoid unacceptable deterioration in image quality. Since the prototype colorectal electronic endoscope described in this report incorporates a 410,000-pixel, high-density CCD, deterioration in image quality during zooming is minimized. Moreover, images can be magnified by a factor of up to 2.0 during both standard and optically magnified examination, permitting clear images of the pit patterns of the colorectal mucosa to be obtained with ease.

### References

[1] Kudo, S., Hirota, S., Nakajima, T. *et al.* Colorectal tumors and pit pattern. *J. Clin. Pathol.* 1994; **47**: 880–885.

[2] Katayama, O., Namiki, K., Ryo, K. *et al.* Colonoscopy using the high-image-quality electronic endoscope TCE-3680MH incorporating a 410,000-pixel CCD. *Medical Review* 1998; **69**: 45–49.

[3] Katayama, O., Okubo, Y., Kato, A. *et al.* Clinical evaluation of electronic zooming with the gastroduodenal electronic endoscope TGI-3000D. *Medical Review* 1994; **55**: 25–29.

[4] Katsu, K., Ichioka, S. and Takemoto, T. The study of endoscopic dyeing method by crystal violet-part 1. *Gastroenterol. Endosc.* 1979; **21**: 1205–1211.

[5] Turell, R. Fiber optic colonoscope and sigmoidoscope, preliminary report. *Am. J. Surg.* 1963; **105**: 133–136.

[6] Classen, M. and Phillip, J. Electronic endoscopy of the gastrointestinal tract, initial experience with a new type of endoscope that has no fiberoptic bundle for imaging. *Endoscopy* 1984; **16**: 16–19.

[7] Tabuchi, M. Endoscopic diagnosis for diminutive colorectal lesions using magnifying electronic endoscope. *Gastroenterol. Endosc.* 1992; **34**: 1993–2002.

[8] Tada, M., Suyama, Y., Tanaka, Y. *et al.* Ultra-magnifying observation of the colon mucosa. *Gastroenterol. Endosc.* 1984; **26**: 49–59.

[9] Kudo, S., Kusaka, N. and Nakajima, T. Minute surface structure of the depressed type early colorectal cancer. *Stomach and Intestine* 1992; **27**: 963–975.

